# The water incident database (WAID) 2012 to 2019: a systematic evaluation of the documenting of UK drownings

**DOI:** 10.1186/s12889-021-11827-0

**Published:** 2021-09-27

**Authors:** Samuel P. Hills, Matthew Hobbs, Michael J. Tipton, Martin J. Barwood

**Affiliations:** 1 Faculty of Health and Social Sciences, Bournemouth University, Bournemouth, BH12 5BB UK; 2 School of Health Sciences, College of Education, Health and Human Development, University of Canterbury, Christchurch, New Zealand; 3 GeoHealth Laboratory, University of Canterbury, Christchurch, New Zealand; 4 The Extreme Environments Laboratory, Department of Sport and Exercise Science, University of Portsmouth, Portsmouth, PO1 2ER UK; 5 International Drowning Researchers’ Alliance, Kuna, ID USA; 6 Department of Sport, Health and Nutrition, Leeds Trinity University, Brownberrie Lane, Horsforth, Leeds, West Yorkshire LS18 5HD UK

**Keywords:** Immersion, Cold water, Accident, Suicide, Injury, Environmental risk factor

## Abstract

**Background:**

Death by drowning is a leading cause of accidental death in the United Kingdom (UK) and worldwide. The World Health Organization (WHO) states that effective documentation of drowning is required to describe drowning frequency and to underpin effective drowning prevention intervention, thus improving the quality of data describing drowning frequency represents a key initiative. The water incident database (WAID) has been used to document UK fatal and non-fatal water-based incidents since 2009. WAID has not undergone a systematic evaluation of its data or data collection procedures to establish if the database meets the WHO requirements. The present study investigated the characteristics of UK fatal drowning incidents and audited current WAID data capture procedures.

**Methods:**

Data for the fatal drowning cases recorded between 2012 and 2019 were reviewed. Descriptive data were generated 1) to describe fatal drownings in the UK’s WAID in this period; 2) a sub-set of drownings were audited i) for completeness of data entry and, based on source documents, ii) for quality of data entry; 3) these processes were used to make recommendations for onward revisions to WAID.

**Results:**

A total of 5051 fatalities were recorded between 2012 and 2019. Drowning was most frequent amongst males aged 35 to 60 years (*n* = 1346), whilst suspected accidents and suicides accounted for 44 and 35% of fatalities. Suicide by drowning was at a peak in the most recent year of data analysed (i.e., 2019; 279 cases) highlighting an urgent need for targeted intervention. Audit part 2i) indicated that 16% of all fields were incomplete, thus indicating potential redundancy, duplication, or the need for onward review. Audit part 2ii) indicated high levels of agreement (80 ± 12%) between audited cases and the ‘true’ WAID entries.

**Conclusions:**

This study confirms WAID as a rigorous, transparent and effective means of documenting UK drownings thereby meeting WHO requirements for data quality; yet future improvements are recommended. Such findings allow researchers and policy makers to use WAID to further investigate UK drowning with a view to improving public safety measures and drowning prevention interventions**.** Observations alongside several expert recommendations have informed a revised version of WAID.

## Background

Globally, drowning accounted for 372,000 lives lost in 2014 and is a leading cause of accidental death in most countries [1]. This death toll is almost two thirds that of malnutrition and over half that of malaria [1]. Drowning is also amongst the ten leading causes of death in children and young people in every region of the World [1], is a primary cause of occupational death and injury, and is a particular problem in middle- and low-income countries [2]. This global figure is thought to represent an underestimation by four or five times due to very limited mechanisms for documenting drowning deaths in many countries [2, 3].

Even in developed countries such as the United Kingdom (UK), drowning is a leading cause of accidental (i.e., unintentional) and intentional death. Indeed, fatal drowning accounts for 400 to 750 deaths, including suicides, each year [4]. Non-fatal water-based incidents are between 20 and 50 times that of the drowning rate although these data are rarely reported [5]. Data from comparable developed countries such as Australia suggest approximately $188 million AUD (~£105.9 million GBP) is spent annually on responding to drowning and water-based incidents, indicating a substantial economic cost to society in addition to the emotional burden associated with each case [6]. In many cases such death and injury can be avoided with evidence-based safety advice and planning to support intervention [1, 7].

Since 2009, the Water Incident Database (WAID) has been used to document UK fatal (referred to as drownings here on) and non-fatal water-related incidents. The database was conceived and constructed by the National Water Safety Forum (NWSF) with the support of the Royal Society for the Prevention of Accidents (RoSPA), who host the database. WAID brings together water-related incident data from a wide range of sources within the UK search and rescue region. In doing so, the database could meet the key initiative of the World Health Organization (WHO) to improve the quality of data describing drowning frequency [1] and may potentially underpin effective drowning prevention interventions [8]. The key aims of WAID are to i) provide insights into levels of risk (including risk acceptability), enabling meaningful comparisons with activities outside the water sector; ii) to supersede the uncoordinated efforts of organisations trying to establish national trends based on limited data of uncertain quality; iii) to produce much higher quality evidence; and iv) to maximise value and minimise aggregate cost of data collection [9]. Data relating to each incident are entered into WAID by water safety agencies (primary data; e.g., Royal National Lifeboat Institution, Her Majesty’s Maritime Coastguard Agency, the National Fire Chiefs Council, the Royal Lifesaving Society & RoSPA) in accordance with pre-defined fields and taxonomies. Presently WAID includes many thousands of entries from several sources relating to each water-based incident, including inquests and has already informed the 2016–2026 UK drowning prevention strategy [9]; which has the aim to reduce drownings by 50% by 2026.

Drowning is a multifactorial phenomenon [10] and it is therefore important that data relating to each water-based incident distinguish between the outcomes and, if possible, the contributory factors that are specific to a given country or event [8]. Establishing these distinctions and contributory factors may enable targeted prevention strategies. For example, it is likely that at least one causal factor in many fatal and non-fatal water-based incidents relates to persons entering the water accidently [4]. Further complications can partly be attributed to the low average annual water temperature around the UK which is between 11 and 13 °C [11]. Such water temperatures are known to evoke the life-threatening cold shock response [12, 13] on immersion which increases the chance of aspirating water to the lung causing asphyxiation precipitated by a loss of respiratory control [12, 13]. Cold water is also one of the attributed reasons that persons often drown within 3 m of the safe refuge of land as a result of swim-failure [14]. Recorded data must also distinguish those circumstances where persons voluntarily enter the water for reasons of recreation (e.g., [15]) where natural causes (such as an underlying health condition; e.g., [16]) and suicide are potential contributors to drowning, the risk of which may be compounded by drug or alcohol intoxication (e.g., [17]). There are also plausible scenarios where a victim forcibly enters the water as part of criminal related activity [18]. Clearly an effective database must comprise of a transparent and unbiased mechanism for documenting and distinguishing between these eventualities. To date WAID processes and data have not been independently assessed to verify the rigour and quality of the collection procedures and outputs.

This study aimed to 1) describe the characteristics of UK fatal drowning incidents, including water related fatalities, that occurred between 2012 and 2019. Thereafter we aimed 2) to audit the procedures of data capture in a two-phase blind audit of a sub-set of drownings i) for completeness of data entry and, based on source documents, ii) for quality of data entry. This study forms the first step in a programme of work planning to undertake epidemiological research with this UK drowning data set. Scientific convention in such onward studies indicates that the data must first be audited [19, 20] to ensure the data quality is fit to address the proposed research questions [21]. In doing so our project represents the first step in scrutinising the database against the WHO’s drowning prevention implementation guide relating to data collection and quality [22]. Also in line with this implementation strategy, we sought 3) to make onward developmental recommendations for inclusions and revisions to WAID to improve the data captured in relation to each fatal and non-fatal drowning event. These recommendations were to inform a second iteration of the WAID database planned for future launch; WAID*v*2.

## Methods

### WAID data set

Specific project approval was granted by the Leeds Trinity University School of Health and Social Sciences research ethics committee (SHSS-2020-03), and a formal data agreement was signed between the authors and RoSPA (the data owner) prior to any data being exchanged. This written agreement explicitly defined the scope of data use, secure storage, and dissemination of related research findings. This study focused only on data relating to fatal incidents recorded by WAID over a pre-defined time period. Accordingly, anonymised WAID data were received for drowning incidents occurring between 1st January 2012 and 31st December 2019, inclusive. After removing any confirmed non-fatal and duplicate cases, the final dataset for this research consisted of 5051 fatalities by drowning that were recorded in WAID.

#### Current WAID data entry procedures

**Fig. 1:**
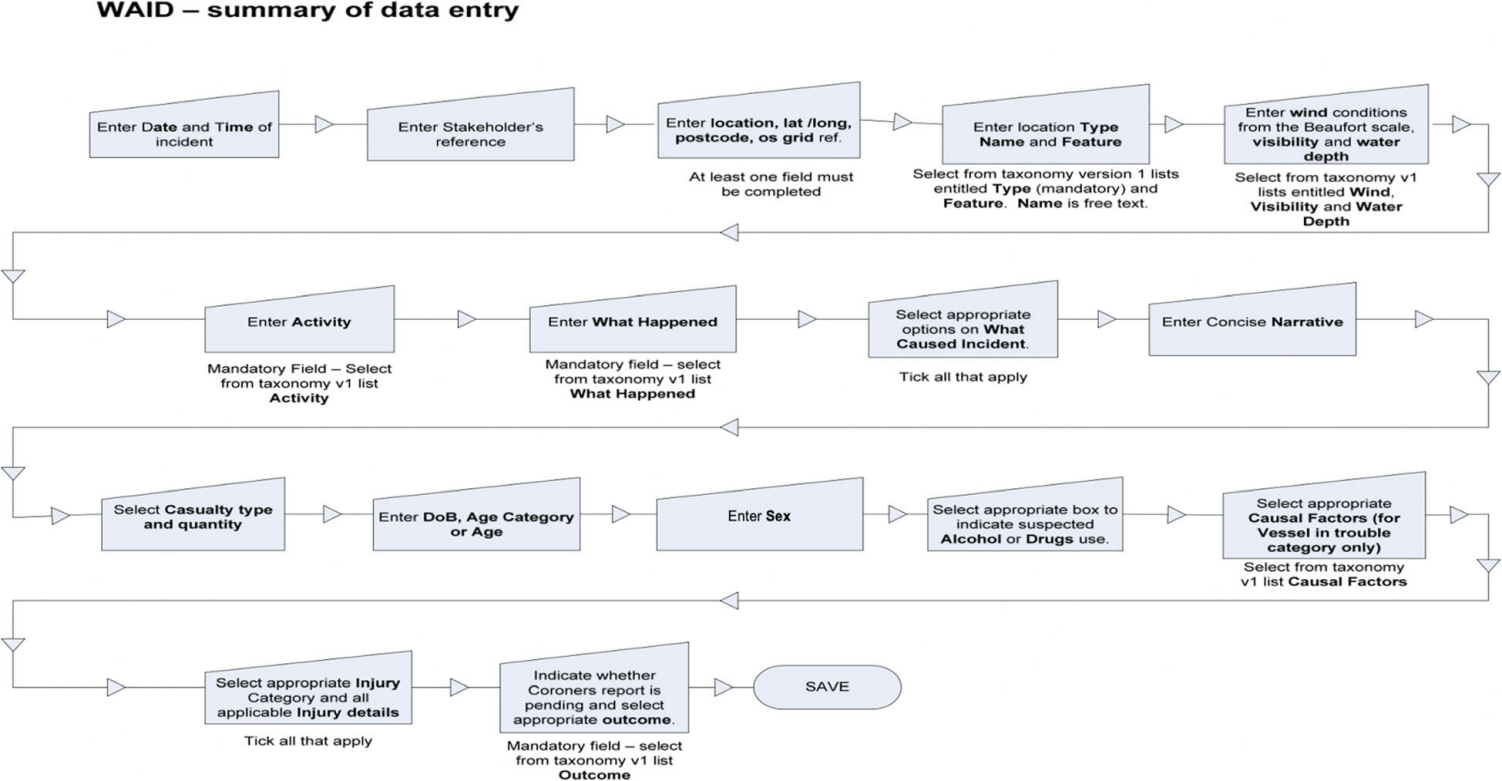
Schematic overview of WAID data entry sequence for each fatal and non-fatal case

**Fig. 2:**
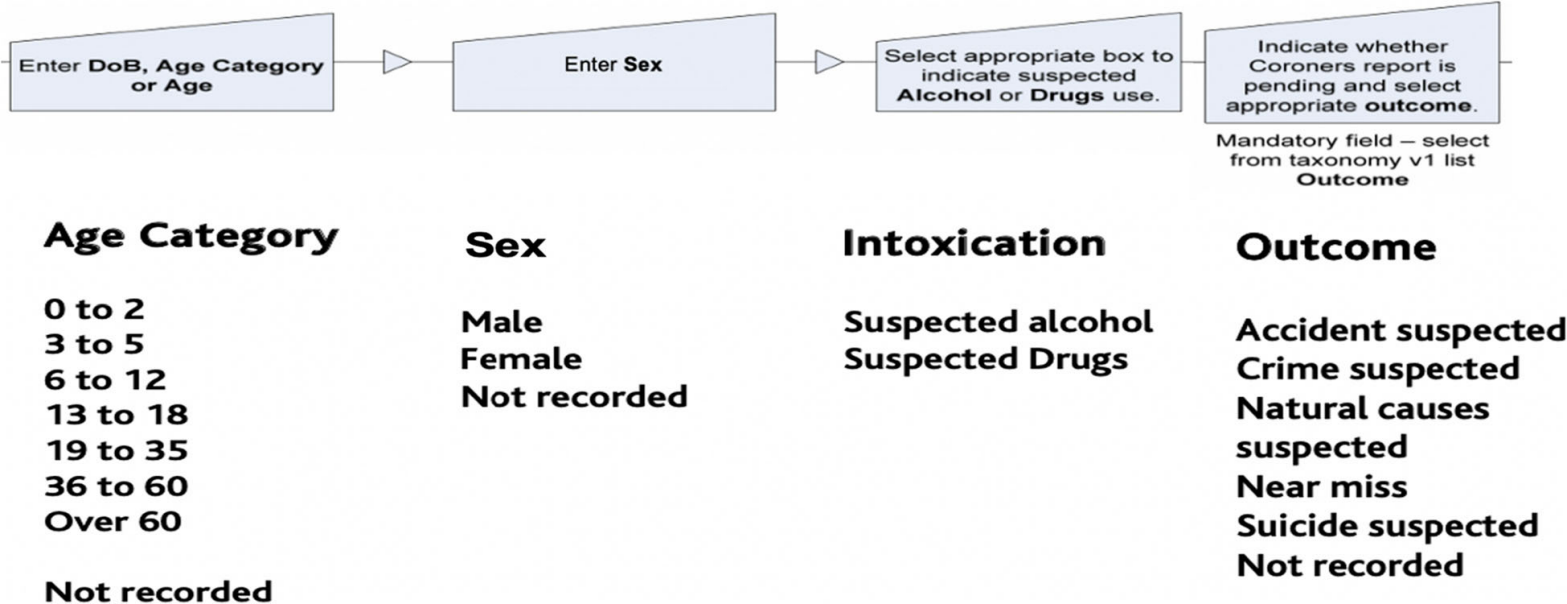
Examples of the sub-sets of the choices available in WAID for the fields of age category, sex, (suspected) intoxication and the mandatory field describing the suspected outcome

Having established a date and time for the incident and a stakeholder reference number (i.e., a reference to the safety, rescue or public organisation responsible for entering the data into the main database), each onward WAID data entry phase requires one or more tick box or narrative entries as part of expanded sub-sections. Table 1 provides operational definitions for the 22 WAID field taxonomies comprising this process and all were present as options throughout the time period of analysis. Figure 1 provides a schematic overview of the sequence followed for incidents entered into WAID including mandatory fields. Figure 2 provides examples of the sub-sets of the choices available for the fields of age category, sex, (suspected) intoxication and the mandatory field describing the *suspected* outcome. The relevant information for each incident is then converted in summarised form, with each row representing a distinct water-based incident and each column representing a WAID field taxonomy.

**Table 1:** Operational definitions for the 22 Water Incident Database (WAID) field taxonomies

Field number	Field name	Operational definition
**1**	**Waidised ID**	Unique identifier number assigned to each case by the Royal Society for the Prevention of Accidents
**2**	**Stakeholder reference**	Identifier number associated with the specific water safety agency entering the case
**3**	**What happened**	Classification of how a person came into difficulty. Alternatively, ‘body recovered’ can be entered
**4**	**Date**	Date and time that an incident or body recovery occurred
**5**	**Activity**	Classification of the action or type activity being undertaken by the victim prior to drowning
**6**	**Postcode**	Postcode at which the incident or body recovery occurred
**7**	**Latitude**	Latitude at which the incident or body recovery occurred
**8**	**Longitude**	Longitude at which the incident or body recovery occurred
**9**	**Ordinance Survey reference**	Ordinance Survey coordinates at which the incident or body recovery occurred
**10**	**Location name**	Name of the location at which the incident or body recovery occurred
**11**	**Location type**	Type of location in which the incident or body recovery occurred
**12**	**Location feature**	Type of feature at or near which the incident or body recovery occurred
**13**	**Wind**	Progressive numerical scale used to indicate the wind conditions at the time of an incident occurring
**14**	**Visibility**	Visibility at the time of an incident occurring
**15**	**Water depth**	Water depth in which the incident or body recovery occurred
**16**	**Age (years)**	Age of the victim in years
**17**	**Age category**	Age category to which the victim belonged
**18**	**Injury**	Severity (fatal, serious, minor, near miss, not recorded)
**19**	**Sex**	The sex of the victim
**20**	**Coroner report**	Whether or not the outcome of a coroner’s report is currently pending
**21**	**Narrative**	A free-text field allowing a brief description of the incident to be entered
**22**	**Intoxication**	Whether or not intoxication by alcohol and/or drugs was suspected or confirmed

### Characteristics of UK fatal drowning incidents 2012 to 2019

The available WAID data were imported into R Studio (*V* 3.6.1; Vienna, Austria) before each of the 22 primary fields were assessed by descriptive analysis. The prevalence of recorded UK drowning cases was quantified overall, by date- (i.e., 2012 to 2019, inclusive), or categories available within WAID and combinations thereof. The number of WAID entries were therefore calculated in relation to the sex of the victim (i.e., ‘male’, ‘female’, ‘not recorded’), each drowning outcome category listed within the available taxonomies (i.e., ‘accident suspected’, ‘not recorded’, ‘suicide suspected’, ‘natural causes suspected’, ‘crime suspected’; see Fig. 2), the age of the victim (i.e., ‘0 to 2 years’, ‘3 to 5 years’, ‘6 to 12 years’, ‘13 to 18 years’, ‘19 to 35 years’, ‘36 to 60 years’, ‘over 60 years’, ‘not recorded’), and whether the victim was suspected to have been intoxicated (i.e., ‘alcohol’, ‘drugs’, ‘alcohol and drugs’, ‘none’).

### Data audit

A two-phase blind audit approach was taken to the WAID data audit. Phase 2i) involved examining the computerised database itself to quantify the completeness of each field and producing summary statistics describing the completeness of data in each of the 22 fields in WAID. Phase 2ii) assessed the reliability of the process of data entry into WAID by reviewing the stored written data (e.g., in the form of media articles, coroners’ reports, accident reports, court proceedings, etc.) that had been originally used to populate the main database.

### Phase 2i): database completeness audit and summary

The available WAID data were again processed using R Studio (*V* 3.6.1; Vienna, Austria) before each of the 22 primary fields were assessed for completeness. To indicate any potential redundancy or duplication of field information in the present iteration of WAID and allow recommendations to be made for subsequent editions of the database (i.e., to meet aim 3), the number of incomplete or missing entries for each field was determined as an absolute value and then as a percentage of the 5051 total cases.

### Phase 2ii) audit of data entry processes

Prior to phase 2ii, data from WAID age categories ‘0 to 2 years’, ‘3 to 5 years’, ‘6 to 12 years’, and ‘13 to 18 years’ were removed as a condition of the ethical approval that was granted to minimise onward emotional trauma to the auditors caused by reading written evidence relating to child and youth drownings. The remaining cases were grouped according to the drowning outcomes of ‘accident suspected’, ‘crime suspected’, ‘natural causes’, ‘not recorded’ and ‘suicide suspected’ before 50 cases, 10 relating to each outcome, were selected for auditing based on their unique identifier (i.e., “Waidised ID”) using a random sampling function in R Studio. All processes hereafter were piloted before finalising the main audit procedures before their commencement. The written reports (e.g., newspaper articles, coroner reports, other media, etc.) originally used to populate WAID for these cases were printed and redacted by a member of RoSPA staff (a person independent to the research team). They oversaw the removal of personal identifier information such as the names, dates of birth, and addresses of any victims or witnesses. Redacted hard copy documents were then equally divided and reviewed, taking alternate cases, by two members of the research team (MB & SH) to establish the reliability of WAID data entry processes. Agreement levels between auditors were ~ 80% at the pilot stage and ~ 77% (grouped mean across auditors) between the auditor entry and the ‘true’ entry latterly confirmed on WAID. It is worthy to note that more than one fatality can be associated with a “Waidised ID” which relate to cases rather than individual persons.

To reflect the typical mode of data entry into WAID, the auditors used the redacted hard copy documents to populate an electronic “dummy” version of WAID fields and taxonomies using the NWSF test site. This site is functionally the same as the main WAID data entry site but populates a separate database used for training. Once all 50 randomly selected cases had been individually considered and entered into the dummy database, auditors’ entries were compared to the true WAID record. The extent of agreement in each case was quantified by assigning each field a value of ‘0’, ‘0.5′, or ‘1’ when entries did not agree, partially agreed (i.e., determined by consensus amongst the research team), or were identical to those listed in WAID. For the fields of latitude and longitude, data were considered to be identical (i.e., a value of 1 was assigned) when dummy and WAID entries were consistent to an accuracy of ≥4 decimal places representing a threshold of 11 m leeway to achieve a value of 1. The agreement between auditor-entered data and WAID data was then calculated in percentage terms for each field and overall. In addition, both auditors documented their subjective experiences of the processes of data entry, agreement and analysis using a notebook. Such information was recorded to assist in providing recommendations for potential improvement in the design and administration of subsequent editions of WAID (to meet aim 3). The phase two audit did not consider the fields of “Waidised ID” and “stakeholder reference” as these fields pertain purely to pseudo-anonymised case identifier information.

## Results

### Characteristics of UK fatal drowning incidents 2012 to 2019

A greater prevalence of drowning was observed in males (a total of 3722 of the 5051 fatalities; ~ 74%) compared with females (1021 cases; ~ 20%), whilst there were 308 (~ 6%) incidents where sex was “not recorded”. The raw count distribution of drowning cases across the age categories outlined in WAID is displayed in Table 2, whereby the most deaths (36%) occurred in 36- to 60-year-olds. When data were expressed relative to the number of years that each age category spanned, the highest drowning rate remained within the 36 to 60 age category (67 p/y).

**Table 2:** Number of Water Incident Database (WAID) recorded drowning cases by age category; *n* = 5051 (cases p/y)

Age category	0 to 2 yrs	3 to 5 yrs	6 to 12 yrs	13 to 18 yrs	19 to 35 yrs	36 to 60 yrs	Over 60 yrs	Not reco-rded
**Total cases** **(cases p/y)**	39 (13)	26 (9)	34 (5)	189 (32)	1138 (67)	1807 (72)	1181(NA)	637 (NA)

WAID age category

*NA* Not applicable

The number of drowning deaths expressed by sex and age group are displayed in Table 3. The most numerous age category for both males and females was 36 to 60 years old indicating a common age for drowning prevalence. When expressed p/y drowning remained most prevalent in females aged 36 to 60 years but changed to the 19 to 35 age category in males. Drug and/or alcohol intoxication was implicated in a total of 820 deaths (16% of total cases), with 604 (74%) of this 820 associated with intoxication by alcohol alone, 104 (13%) cases associated with drugs alone, and 112 (14%) drownings associated with a combination thereof.

**Table 3:** Number of Water Incident Database (WAID) recorded drowning cases by sex and age category; *n* = 5051 (cases p/y)

Age category (category range)	Sex	Total cases (cases p/y)
0 to 2 years	Female	11 (4)
	Male	27 (9)
	Sex not recorded	1 (0)
3 to 5 years	Female	6 (2)
	Male	19 (6)
	Sex not recorded	1 (0)
6 to 12 years	Female	13 (2)
	Male	21 (3)
	Sex not recorded	0 (0)
13 to 18 years	Female	53 (9)
	Male	184 (31)
	Sex not recorded	19 (3)
19 to 35 years	Female	167 (10)
	Male	949 (56)
	Sex not recorded	22 (1)
36 to 60 years	Female	414 (17)
	Male	1346 (54)
	Sex not recorded	47 (2)
Over 60 years	Female	301 (NA)
	Male	854 (NA)
	Sex not recorded	26 (NA)
Age not recorded	Female	83 (NA)
	Male	346 (NA)
	Sex not recorded	208 (NA)

*NA* Not applicable

**Fig. 3:**
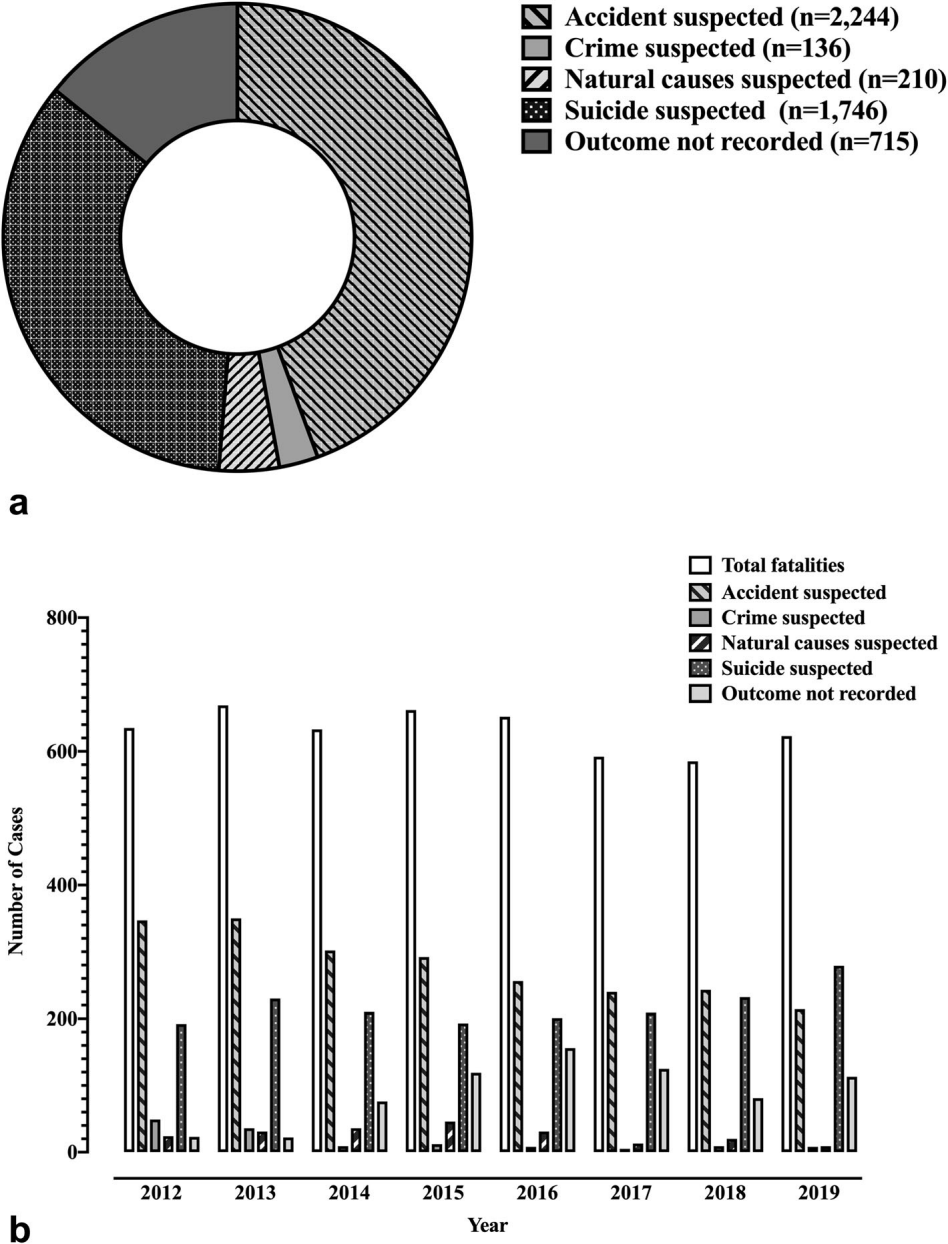
Number of Water Incident Database (WAID) recorded drowning cases by drowning outcome (**a**) and calendar year (**b** *n* = 5051*)*

The distribution of the 5051 cases across each drowning outcome category is expressed in Fig. 3a, whilst Fig. 3b shows the number of WAID recorded cases for each year of the study period across the five downing outcomes. Approximately 44 and 35% of WAID entries recorded over the study period were suspected to be results of accidents and suicides, respectively. The greatest number of annual fatalities were recorded in 2013, while the least occurred in 2018.

### Phase 2i): database completeness audit and summary

Table 4 shows the completeness of the 22 WAID fields examined in phase one of the audit. Several fields were populated for all or most of the 5051 cases such as latitude and longitude, whereas some fields (e.g.,“visibility” and “water depth”) were largely incomplete. From a total of 111,122 potential field entries (i.e., 22 fields from 5051 cases), 84% were complete.

**Table 4:** Number and percentage of missing values for each Water Incident Database (WAID) field taxonomy; *n* = 5051

Field number	Field name	Total missing cases	Missing cases (%)
**1**	**Waidised ID**	0	0.0
**2**	**Stakeholder reference**	14	0.5
**3**	**What happened**	24	0.5
**4**	**Date**	0	0.0
**5**	**Activity**	6	0.1
**6**	**Postcode**	544	10.8
**7**	**Latitude**	0	0.0
**8**	**Longitude**	0	0.0
**9**	**Ordinance Survey reference**	492	9.7
**10**	**Location name**	28	0.6
**11**	**Location type**	24	0.5
**12**	**Location feature**	41	0.8
**13**	**Wind**	4606	91.2
**14**	**Visibility**	4492	88.9
**15**	**Water depth**	4917	97.3
**16**	**Age (years)**	734	14.5
**17**	**Age category**	637	12.6
**18**	**Injury**	0	0.0
**19**	**Sex**	308	6.1
**20**	**Coroner report**	0	0.0
**21**	**Narrative**	0	0.0
**22**	**Intoxication**	0	0.0

### Phase 2ii) audit of data entry processes

The 50 case records analysed described a total of 58 fatalities. The overall level of agreement between auditor dummy entries and the true WAID entries was 80 ± 12%; 79 ± 13% and 81 ± 11% for each auditor; see Table 5. Fields such as ‘injury’, ‘water depth’, and ‘wind’ showed the greatest agreement (> 95%). Conversely, ‘Ordinance Survey reference’ and ‘what happened’ had agreement values < 60%. The auditors’ subjective experiences of undertaking the audit suggested that establishing the identifier characteristics of the casualty (e.g., age and sex) was relatively straightforward, whereas specific details about the incident such as “what happened” were often more difficult to ascertain. Frequently, a lack of evidence to suggest other possibilities meant that “body recovery” was concluded for the ‘what happened’ field.

**Table 5:** Percentage agreement between auditor-entered dummy cases and true Water Incident Database (WAID) entries for each field taxonomy; *n* = 50 cases

Field number	Field name	Agreement (%)
1	What happened	59
2	Date	69
3	Activity	71
4	Postcode	71
5	Latitude	76
6	Longitude	65
7	Ordinance Survey reference	55
8	Location name	87
9	Location type	82
10	Location feature	66
11	Wind	96
12	Visibility	91
13	Water depth	96
14	Age (years)	88
15	Age category	96
16	Injury	100
17	Sex	91
18	Coroner report	64
19	Outcome	80
20	Intoxication	94

We make onward developmental recommendations for WAID*v2* (i.e. to address aim 3) in the discussion.

## Discussion

This study sought to describe the characteristics of UK fatal drowning incidents that occurred between 2012 and 2019 and to establish the completeness of WAID across the fields currently included in the database. Thereafter we aimed to audit the procedures of data capture relating to a sub-set of drowning cases in WAID and to examine the agreement between the extant data and the data generated by a blind audit. In doing so, this study has been able to establish whether the database could meet the key initiative of the WHO to improve data quality describing drowning frequency [1]. Our findings show that the number of documented UK drownings remained between 585 (in 2018) and 669 (in 2013) deaths per year for each year from 2012 to 2019, inclusive. Moreover, the fact that males most frequently drowned during this time period (i.e., male deaths comprising approximately 74% of the recorded fatalities, acknowledging that the victim’s sex was not recorded for a further 6% of the sample), is a finding broadly consistent with other developed countries; Australia 78%; Canada 81%; New Zealand 82% [8]. During the audit phase of the study we were able to establish a high level of completeness of most fields in WAID indicating the effectiveness of the database in capturing key characteristics relating to water related fatalities and drowning. We were also able to establish that the stored written evidence associated with each case enabled a high level of agreement to be achieved between auditor and true WAID entry by reading the case details and following the procedures of data entry. This study therefore confirms WAID as a rigorous, transparent and effective means of documenting UK drownings; nevertheless further development of WAID is required. Very few studies have undertaken an independent audit of drowning database entry procedures and reported the findings in the open scientific literature [3, 8]. Collectively, these findings now legitimise our intentions to undertake a programme of research using this UK drowning dataset.

Drowning in persons up to the age of 18 accounted for around 5% (262) of total deaths considered in the present study; these national data are comparable to historic, regional data which show drowning as a leading cause of accidental death in children and adolescents [23]. Persons aged 36 to 60 saw the highest frequency of drownings and these comprised of mostly males when considered by sex. Males tend to take greater risks on or around water and intoxication by alcohol or drugs is known to exacerbate this risk taking [24]. Indeed, alcohol was suspected to be implicated, whether solely or in combination with drugs, in a total of 16% of the fatal cases reviewed in the present study. This is low compared to Australia (25.8%) and Canada 36%. Variation in data collection procedures between countries may account for these differences and there remains a need for consistency between countries when collecting drowning data [8].

Approximately 44% of the cases reviewed in the descriptive phase one audit were documented as ‘accident suspected’. The next most numerous outcome category after ‘accident suspected’ was ‘suicide suspected’ (35% of cases). The WHO data set and drowning data from other countries have tended not to focus on intentional drownings (e.g., 3) because no reporting sub-categories existed until recently in the International Statistical Classification of Diseases and Related Health Problems [ICD] codes for suicide by drowning [3] to additionally document intentional drowning. The eleventh edition of the ICD has recognised and addressed this imbalance [25]. The reporting of such data is important as these data provide a more complete picture of drowning fatalities [26]. Observations from the present data set indicate that, acknowledging that sex was not recorded for a further 188 suspected suicides, a higher proportion of female drownings were recorded as “suicide suspected” (44% of female drownings and 30% of male drownings between 2012 and 2019). Whilst not directly comparable, publicly available UK data for 2018 report 4.4% of suicide deaths were by drowning in females compared to 3.8% in males [27]. Collectively, it is of significant concern that suicide by drowning was at a peak in the most recent year of data analysed in the present data set (i.e. 279 cases) highlighting an urgent need for targeted intervention.

### Recommendations for drowning database development

There currently exists no definitive guide to drowning data collection and coding variables [8]. Consequently, we sought to use our procedures to make onward developmental recommendations for inclusions and revisions to WAID (and other databases) to improve the data captured in relation to each fatal and non-fatal drowning event. The recommendations were reached in consultation with RoSPA, on the basis of the auditors’ shared experiences of undertaking the audit, alongside published evidence and the experience of the research team. The recommendations relate primarily to documenting additional factors relating to drowning events and improving the clarity of existing factors considered as part of WAID that may substantially influence an individual’s likelihood of drowning and subsequently inform prevention strategies. These observations are being made openly available in order to inform the practices of other researchers and drowning databases. To avoid potential redundancy of the suggested fields, the value of including these variables must be considered in light of their feasibility and viability for documentation. Observation of the data relating to data completeness (i.e., aim 2ii) have also informed these suggestions). Recommendations for potential inclusion within WAID*v2* were as follows:

i) **Estimated water temperature and water conditions.** It is known that low water temperatures are linked to the magnitude of the life-threatening cold shock response [12] with lower temperatures linked to increased likelihood of drowning [13, 28]. Accordingly, it would be valuable to include a default (rather than optional) estimate of water temperature and water conditions to examine the role these variables play in future WAID cases. Moreover, a water temperature of 6 °C or below has been linked to extended underwater survival time whilst submerged which is also linked to search and rescue (SAR) duration [29]. Therefore, knowledge of water temperature in drowning and non-fatal water accident cases may enable decision-making models to be verified or refined that underpin SAR. A plausible option for populating the database may be to triangulate “live” measured data or data derived from seasonal estimates per given body of water particularly in countries where cold water is a seasonal threat [30, 31].
ii) **Estimated air temperature.** In circumstances where persons voluntarily enter the water the extant air temperature may partially underpin the decision to do so [30]. Data from Canada indicate ambient air temperatures exceeding 30 °C increased the likelihood of outdoor drowning by 69%. Fralick et al. [32] also showed that drowning risk in all age groups and sexes increased with increasing ambient temperature but to the greatest extent in males. Given that coastal and inland water temperatures tend to be lowest during the Spring [11] and high ambient temperatures are plausible at this time, knowledge of the extant air temperature at the approximate date and time of water entry may enable proactive drowning prevention interventions based on weather forecasting. A default entry to WAID*v2* with Triangulation of “live” or recorded data may be a viable option.
iii) **Behavioural factors in drowning.** Drowning is a multifactorial event that may include a significant behavioural component [33]. The inclusion of measures that document behavioural factors may provide valuable information about the behaviours that precede drowning such as visitation of water sites for recreation [34]. For example, it is plausible that drowned victims may travel short or long distances for reasons of leisure to access the water environment. In the case of the latter, a transition may take place from a low drowning risk and warning environment (e.g., a city) into one that carries far greater risk (e.g., coastal or river environments). If proven, proactive drowning prevention interventions could target the point of origin of the potential victim if the behavioural pattern is established. Including simple measures such as the drowning victim’s postcode (where available) may enable targeted intervention according to expected behaviours. Moreover, this variable can also enable the victim’s social demographic to be considered as an influential factor similar to other health conditions [20].
iv) **Ethnicity.** Presently we have only a limited indication of drowning prevalence in minority ethnic groups in the UK. It is plausible that drowning risk or incidence may be higher for social or cultural reasons and targeted intervention based on examining this possibility may be valuable. For example data from Canada, another country where cold water is an influential factor in drowning, indicate the age-standardized drowning rate is significantly higher among men of Asian, African, or Hispanic ethnicity compared to men of Greater European ethnicity and for women of Asian, African, or Hispanic ethnicity compared to women of Greater European ethnicity [35]. Manual (i.e., it is not a mandatory sub-category) entry of this variable currently exists in WAID. Including this variable will ensure WAID aligns with the 11 recommended core variables suggested for effective between-country collection of drowning data [8].
v) **Clothing worn.** We have previously suggested that air trapped between clothing layers in normal seasonal clothing assists short term buoyancy and therefore flotation [4]. Moreover, protecting the skin from rapid skin cooling reduces the extent of the cold shock response [12] and it has been reported that 50% of persons who enter water unintentionally are clothed and drown within 3 m of safe refuge [4]. Knowledge of the extent of clothing worn in cases of drowning may establish the differing role that clothing plays in drowning case outcomes.
vi) **Establishing a flotation factor.** Our laboratory studies have shown variability in flotation capability between individuals [4]. We suggested the ability to float may impact upon the likelihood of drowning [4]. Yet, factors such as body density, lung volume, clothing and water temperature have all been suggested to impact upon flotation capability [36] and could be derived from entries on WAID. Identifying flotation capability as an influential factor in drowning may underpin the educational basis for focussing on the skillset to achieve floating as part of learn to swim and survival skills.
vii) **Separate data for drowning event and recovery.** With recommendations i) to iii) in mind, future recorded data should (where possible) try to distinguish between data available at the time of water entry (date/time of drowning if known) and that derived from the time of body recovery. Many of the WAID phase two audited cases included information from the time of recovery where some of the key variables (e.g., air temperature) may have changed significantly between the time of water entry and body recovery.
viii) **Point of entry to emergency care.** In cases where the immersed victim is rescued alive, the emergency response time and proximity of the required emergency care may be critical in determining the outcome. Documenting the details around the point of entry to emergency care (if completed) or the proximity of the nearest emergency care to the water location may help contextualise the importance of the transition time to receiving medical support; this inclusion would also raise the possibility of Utstein style reporting on the drowning case should it include resuscitation attempts [37].
ix) **Revision to nomenclature.** Lastly, the term “suspected outcome” was used throughout this paper to categorise drownings in line with the structure of WAID. Whilst these outcomes are “suspected” they may be mis-leading as they imply certainty of detail when the evidence is not always substantial or available. Redefining these categories by intent such as unintentional (e.g. accidental, natural causes), intentional (suicide, homicide) and undetermined would help align WAID*v2* more closely to epidemiological categorisation practices [25].

This study is not without limitation. Whereas the procedures underpinning the phase two data audit were standardised between auditors and pilot testing was conducted before the main audit, the persons involved were not experienced users of WAID. It is therefore plausible that some of the audited entries were subject to judgement error on the part of the auditor. However, as further standardised training (similar to the in-house training provided by RoSPA to those individuals responsible for populating WAID) is likely to improve agreement with the true WAID case, it is reasonable to suggest that the present study probably represents a conservative estimate of the agreement. Secondly, we were only able to audit 50 cases (58 fatalities) in phase two, a sample comprising approximately 1% of the database and excluding child and adolescent drownings. It is plausible that the effects we report in this fraction of the data do not reflect the wider picture or that seen in the non-fatal cases and that such observations are restricted to adult drownings only. Nevertheless, the randomly selected sample of cases examined a complete range of all of the recorded WAID drowning outcomes, was completed independently of RoSPA, and required a significant outlay of time, manpower and resource. Future studies including data collected after 2019 may seek to verify our observations.

## Conclusions

In summary, this study sought to describe the characteristics of UK fatal drowning incidents that occurred between 2012 and 2019 and to audit the processes and data stored in WAID. In doing so we are able to conclude that the database meets the key initiative of the WHO to improve data quality describing drowning frequency [1]. However, revisions and potential improvements to WAID are necessary including considering including variables that describe pre-drowning behaviour and distinguishing between the environmental conditions at the time of water entry from the time of body recovery. The data in WAID now appear suitable to be considered for onward study to address the often-neglected public health issue of drowning [3]. This programme of work can now progress to step two of the WHO’s drowning prevention implementation guide with the aim of identifying drowning risk factors [22].

## Data Availability

The dataset describing the individual drowning cases upon which this manuscript is based cannot be made available. The sensitive nature of the data meant that non-disclosure was a condition of the signed data agreement and granting of ethical approval. An anonymised annual report of WAID data at the drowning cohort level is available from the NWSF https://nationalwatersafety.org.uk/waid/annual-reports-and-data/

## Abbreviations

ICD: International Statistical Classification of Diseases and Related Health Problems
NWSF: National Water Safety Forum
RoSPA: The Royal Society for the Prevention of Accidents
UK: United Kingdom
WAID: Water Incident Database
WHO: World Health Organisation
